# Postural habits and lifestyle factors associated with adolescent idiopathic scoliosis (AIS) in China: results from a big case–control study

**DOI:** 10.1186/s13018-022-03366-0

**Published:** 2022-10-29

**Authors:** Jingfan Yang, Sizhe Huang, Mengyuan Cheng, Weiqing Tan, Junlin Yang

**Affiliations:** 1 Xinhua Hospital Affiliated to Shanghai Jiao Tong University School of Medicine, Shanghai, China; 2 Health Care Centre for Primary and Secondary Schools of Zhongshan Municipality, Zhongshan, China; 3 University of North Carolina at Chapel Hill Project-China, Guangzhou, China; 4 Health Promotion Centre for Primary and Secondary Schools of Guangzhou Municipality, Guangzhou, China

**Keywords:** Adolescent idiopathic scoliosis, Risk factor, Epidemiology, AIS, Screening, Postural habits, Lifestyle factors

## Abstract

**Background:**

Adolescent idiopathic scoliosis (AIS) is the most prevalent type of scoliosis affecting children between the ages of 10–16 years. However, risk factors for AIS, particularly the modifiable ones, are still largely unknown. This study aims to investigate the associations of lifestyle and social environment factors with AIS in Chinese schoolchildren.

**Methods:**

This is a matched case–control study based on survey data collected from school-based scoliosis screening program. We used conditional logistic regression models to describe the relative risk of AIS incidence for each variable in the analyses. To examine the independent effect of each factor on developing AIS, a multivariate conditional logistic regression was conducted and odds ratios (ORs) were adjusted for age and other significant variables.

**Results:**

Overall, 2538 participants from 49 schools were included in this study, comprising 1269 AIS cases and 1269 controls. Mean age of the study population was 13.4 years ± 1.06 (range 10–18). One thousand five hundred and fifty (61.1%) of the study subjects were girls. After adjusting for other significant factors, inappropriate desk heights, either too low (OR = 1.40, 95% CI 1.04–1.90) or too high (OR = 1.61, 95% CI 1.09–2.38), standing with anterior pelvic tilt (OR = 2.73, 95% CI 1.41–5.28), and sleeping on the right side (OR = 1.38, 95% CI 1.00–1.91), remained associated with elevated AIS risks. In contrast, sitting normally and classroom sitting positions change regularly were associated with lower odds of AIS. The adjusted ORs were 0.69 (95% CI 0.50–0.96) for sitting normally, and 0.72 (95% CI 0.53–0.98) for sitting positions change.

**Conclusions:**

This is the first study to address the associations between desk heights and AIS and showed inappropriate desk heights were related to increased AIS risks. To protect school children from developing AIS, stakeholders are advised to consider introducing height-adjustable desks in the class, changing students’ sitting positions in the classroom on a regular basis, and implementing educational programs to help students maintain correct sitting postures.

## Background

Adolescent idiopathic scoliosis (AIS) is a structural deformity of the spine characterized by a lateral spinal curvature of ≥ 10° in the coronal plane [1]. It is the most common scoliosis type affecting children aged 10–16 years, with an estimated global prevalence of 1–3% [2–4]. Scoliotic curves can progress rapidly during growth spurt, and severe AIS may result in poor prognosis such as cardiopulmonary compromise, physical deformity and psychosocial disturbances [5–7]. Common treatments for patients whose curvatures do not exceed 40° or 45° Cobb, including bracing and exercises, are in nature attempts to halt curve progression and alter its natural history. Therefore, early identification of AIS is essential because it enables early intervention to delay curvature progression, lower the likelihood of spinal fusion, and enhance the short- and long-term health outcomes of patients.

However, even though risk factors for AIS are crucial in its early detection, previous studies mostly focused on the etiologic causes of AIS [8–13], including genetic, hormonal, or neuromuscular abnormalities, etc., or only on a specific subgroup of AIS at-risk population [14–16], such as swimmers, ballet dancers and schoolgirls, leaving relationships between posture, lifestyle and social environment factors and AIS understudied and undetermined. In a study [17] conducted by Zheng et al. in Wuxi, China, a total of 11,024 primary school students were enrolled for determining prevalence and determinants of idiopathic scoliosis, and the researchers found that female, use of single-shoulder bags, and more time using a computer were AIS predictors for the studied population. However, small number of AIS cases (*n* = 11) and variables included in the scoliosis determinants assessment limited the generalizability and comparability of its findings, despite the large sample size for AIS prevalence analysis. Additionally, a large-scale (*n* = 2759) cross-sectional study conducted by Watanabe et al. [14] provided vigorous findings about the AIS risk factors for local schoolchildren, namely classical ballet training, family history of scoliosis and low body mass index (BMI); however, study population containing only schoolgirls discourages it to reliably describe AIS risk factors for all at-risk schoolchildren.

To bridge the research gap, this study aimed to evaluate the relationships between the presence of scoliosis and a number of patient characteristic, postural habits and lifestyle factors in Chinese school setting. This information will be used to guide future studies on identifying high-risk individuals for targeted scoliosis screening and implementing preventive measures for AIS at schools or clinics. And we hypothesized that postural habits and lifestyle factors like inappropriate sitting posture and long time spent sitting were associated with elevated AIS risks.

## Methods

### Study design and participants

This case–control study was embedded in the 2015–2017 school-based scoliosis screening program for adolescents in Guangzhou Municipality, China, in which more than 295,650 schoolchildren from 476 secondary schools were screened for AIS. Participants were recruited between September 2016 and July 2017, from randomly selected participating schools without special considerations for regions or grades. Cases were schoolchildren with positive screening results. Controls were those who tested negative and were randomly matched with cases in a 1:1 ratio for sex and school. Participants diagnosed with congenital scoliosis and neuromuscular scoliosis were excluded from this study.

### Screening process

Detailed screening process has been previously described [18]. The school scoliosis screening began with a visual inspection in the upright position for spine alignment, asymmetry of the shoulder and breasts, scapula prominence, etc*.*, followed by a forward bending test (FBT). If any trunk rotation deformity was noted by the examiner, a scoliometer would be employed to quantify the angle of trunk rotation (ATR). Any child with a scoliometer reading of more than 5° or with 2 or more significant clinical signs would be advised to visit specialized physicians for further evaluation, and a referral letter recommending a diagnostic standing posteroanterior radiograph of the spine would be sent to the child’s parents or guardians.

### Questionnaire

Structured questionnaires were administered in the same manner in cases and controls. Students received a face-to-face interview immediately after being screened, and they were asked to complete a questionnaire, before receiving their screening results or any recommendations for further follow-up. The questionnaire contained 31 questions, and was designed to collect information related to students’ demographics, lifestyle and social environmental characteristics, such as age, sex, physical activity time, standing and sitting postures, etc. The questionnaire was adapted from the Back Pain and Body Posture Evaluation Instrument (BackPEI) and was further developed by two orthopedic surgeons specializing in the treatment of scoliosis (Z.F.Huang, J.L.Yang), together with several experienced screen examiners after extensive discussion. Variables included were selected based on previous study findings, BackPEI and input from clinicians.

### Measurements of outcome and risk factors

AIS is diagnosed when a lateral spinal curvature of or greater than 10° was detected on a coronal radiographic image. However, since it was not feasible to obtain whole spine radiographic images of healthy schoolchildren, in this study, we took school screening results as outcome measurement. The screening protocol used in this study was the same standardized one used in another study [18] that we have done before, where of 6537 children who were referred for radiograph examinations after screening, 5125 had confirmed diagnosis of AIS. We would expect a similar accuracy rate for the screening in this study as in the previous one, which was about 80%.

Reported risk factors that may be associated with AIS, such as age, sex, family history, physical exercise or participation in sports, time spent seated, body posture while sitting, sleeping, reading, etc. were included and measured in the questionnaire. The Back Pain and Body Posture Evaluation Instrument (BackPEI) questionnaire was translated into Chinese and used for assessing postural habits with minor modification in wording and options to better reflect Chinese schoolchildren’s general living situations. Pictures demonstrating body postures or types of schoolbags were provided for children to compare to when assessing themselves. In the study questionnaire, standing posture was categorized as normal, standing with anterior pelvic tilt and standing with hunchback. Sitting posture was divided as follows: normal (up-straight), lean forward, lean left, lean right, sitting with chest on the desk, sitting with crossed legs and others. Sleeping posture was categorized into supine, prone, left lateral and right lateral. School and home desk heights were defined as appropriate, lower or higher when they were equal to, lower or higher than their forearm when participants were seated up-straight with their elbows in 90° angle. Classroom lighting was graded by the students as appropriate, bright or dark. Eyesight condition was divided into normal and myopia (near sighted), and the latter should be diagnosed by ophthalmologists. Four categories, namely backpack on the back, backpack on the front, shoulder bag and cross-shoulder bag, were used to describe participants’ schoolbag wearing habits. Shoe wear patterns were divided into three categories, namely no difference between shoes, left shoe wears harder and right shoe wears harder. So was students’ general health status for the previous year, which was categorized as fair, good and poor. Time spent on physical activity per week was measured in minutes. Height increment was defined as the height participants gained in the previous year and was measured in millimeter. Posture improvement suggestions, postural education in school, napping on school desk, extracurricular classes, low back pain and sports participation were all processed as binary variables (Yes/No).

### Statistical analysis

Given the case–control design of our study, we used conditional logistic regression models to describe the relative risk of AIS incidence for each variable in the analyses. Odds ratios (ORs) and accompanying 95% confidence intervals (CIs) were presented to describe the associations. To examine the independent effect of each factor on developing AIS, a multivariate conditional logistic regression was conducted and ORs were adjusted for age and other significant variables.

All statistical tests of hypothesis were two-sided, and statistical significance was set at 0.05. Statistical analyses were all performed using Stata 15 (StataCorp, College Station, Texas, USA).

## Results

### Participants’ characteristics

Overall, 2538 participants from 49 schools were included in this study, comprising 1269 cases of AIS and 1269 healthy controls. Descriptive characteristics of cases and controls are reported in Table 1. Mean age of the study population was 13.4 years ± 1.06 (range 10–18). One thousand five hundred and fifty (1550, 61.1%) of the study subjects were female, and 988 (38.9%) were male.

**Table 1:** Characteristics of the study population and the two scoliosis groups

Characteristics	Overall	AIS	Non-AIS
	*n* = 2538	*n* = 1269	*n* = 1269
Age, mean (SD), yr	13.4 (1.06)	13.6 (1.04)	13.3 (1.05)
*Sex*			
Female	1550	775 (61.1)	775 (61.1)
Male	988	494 (38.9)	494 (38.9)
*Standing posture*			
Normal	1825	830 (67.7)	995 (80.7)
Anterior pelvic tilt	148	97 (7.9)	51 (4.1)
Hunchback	486	299 (24.4)	187 (15.2)
Sitting posture*			
Straight	717	281	436
Leaning forward	1142	606	536
Leaning left	657	379	278
Leaning right	441	241	200
Chest touching desk	520	257	263
Cross-legged	709	360	349
Others	106	52	54
*Sleeping posture*			
Supine	926	426 (34.8)	500 (41.0)
Prone	194	74 (6.0)	120 (9.8)
Left lateral	734	397 (32.1)	340 (27.9)
Right lateral	591	332 (27.1)	259 (21.3)
*Ever received posture improvement suggestions*			
Yes	1508	779 (63.9)	729 (59.6)
No	934	440 (36.1)	494 (40.4)
*Ever received postural education at school*			
Yes	1652	812 (66.7)	840 (68.8)
No	787	406 (33.3)	381 (31.2)
*Napping on school desk*			
Yes	1707	835 (67.2)	872 (70.1)
No	780	408 (32.8)	372 (29.9)
School desk height			
=	1137	496 (42.1)	641 (53.5)
<	933	508 (43.1)	425 (35.5)
>	307	174 (14.8)	133 (11.1)
*Home desk height*			
=	1100	482 (41.2)	618 (52.5)
<	824	447 (38.2)	377 (32.0)
>	424	242 (20.7)	182 (15.5)
Eyesight*			
Normal	984	474	510
Myopia	1235	624	611
*Regular change of sitting position in classroom*			
No	1608	782 (66.1)	826 (69.9)
Yes	757	402 (33.9)	355 (30.1)
*Classroom lighting*			
Appropriate	2056	1031 (85.6)	1025 (84.7)
Bright	258	118 (9.8)	140 (11.6)
Dark	101	56 (4.7)	45 (3.7)
*Schoolbag use habits*			
Backpack on the back	2163	1038 (93.4)	1080 (91.1)
Backpack on the front	81	36 (3.1)	45 (3.8)
Shoulder bag	66	29 (2.5)	37 (3.1)
Cross shoulder bag	36	12 (1.0)	24 (2.0)
*Shoe wear patterns*			
No difference	2039	1018 (85.3)	1021 (86.5)
Left shoe wears harder	155	81 (6.8)	74 (6.3)
Right shoe wears harder	181	95 (7.9)	86 (7.3)
*Extracurricular classes*			
No	856	414 (34.7)	442 (36.6)
Yes	1543	778 (65.3)	765 (63.4)
PE class (per week)			
None	11	8 (0.7)	3 (0.3)
1	109	60 (5.0)	49 (4.1)
2	654	320 (26.7)	334 (28.0)
3	1483	739 (61.6)	744 (62.3)
> 3	136	72 (6.0)	64 (5.4)
Average physical activity time (per week, *min*)	44.3 (41.3)	41.6 (40.8)	47.0 (41.6)
Annual height increment, mean (SD), *mm*	51.2 (16.6)	50.3 (16.9)	52.2 (16.2)
*General health status*			
Fair	804	433 (36.4)	371 (31.3)
Good	1515	730 (61.4)	785 (66.1)
Poor	57	26 (2.2)	31 (2.6)
*Low back pain*			
No	1884	954 (74.9)	930 (73.1)
Yes	662	319 (25.1)	343 (26.9)
Sports participation (after school)			
*Badminton*			
No	1725	888 (69.8)	837 (65.8)
Yes	821	385 (30.2)	436 (34.3)
*Basketball*			
No	2040	1024 (80.4)	1016 (79.8)
Yes	506	249(19.6)	257 (20.2)
*Running*			
No	1307	651 (51.1)	656 (51.5)
Yes	1239	622 (48.9)	617 (48.5)
*Swimming*			
No	2031	1052 (82.6)	979 (76.9)
Yes	515	221 (17.4)	294 (23.1)
*Dancing*			
No	2377	1185 (93.1)	1192 (93.6)
Yes	169	88 (6.9)	81 (6.4)
*Mountain climbing*			
No	2297	1169 (91.8)	1128 (88.6)
Yes	249	104 (8.2)	145 (11.4)
*Ping pong*			
No	2331	1177 (92.5)	1154 (90.7)
Yes	215	96 (7.5)	119 (9.4)
*Morning exercises*			
No	2180	1106 (86.9)	1074 (84.4)
Yes	366	167 (13.1)	199 (15.6)
*Football*			
No	2361	1186 (93.2)	1175 (92.3)
Yes	185	87 (6.8)	98 (7.7)
*Stroll*			
No	2525	1266 (99.5)	1259 (98.9)
Yes	21	7 (0.6)	14 (1.1)

Number (%) were presented unless stated otherwise

*Multiple-choice questions

There were statistically significant differences in the patterns of standing, sitting and sleeping postures (*p* < 0.01) with, most notably, only 67.7% of the AIS group reported a normal standing posture, while that figure for the non-AIS group was 80.7%. Larger percentages of people received posture improvement suggestions in the AIS group than in the non-AIS group (63.9% vs. 59.6%), while the percentage of participants received posture education at school was slightly lower in the AIS group (66.7% vs. 68.8%). Compared with non-AIS participants, fewer AIS participants napped on school desk (67.2% vs. 70.1%) during lunch breaks or had a desk with an appropriate height at school (42.1% vs. 53.5%) or at home (41.2% vs. 52.5%). The average height increment in the previous year for scoliotic participants was 50.3 mm ± 16.9, slightly lower than that for the non-scoliosis students, which was 52.2 mm ± 16.2. Besides, according to the self-reported estimations of their daily exercises condition, AIS group had lower participation rates for sports such as badminton, basketball, swimming, mount climbing, ping-pong ball, morning exercise, football and stroll.

No significant differences were found between the two groups in terms of the amount of time spent on extracurricular classes, proportions of participants with normal sight condition, the way to carry their school bags during daily commute and general health status.

### Factors associated with AIS

Associations of lifestyle and social environment factors with AIS are reported in Table 2. Univariate conditional logistic regression analyses (Table 2) showed that the risk of AIS increased by 28% when students grew 1 year older (OR = 1.28, 95% CI 1.18–1.38), and the association remained (OR = 1.17, 95% CI 1.03–1.34) after adjusted for other significant factors in the multivariate analyses.

**Table 2:** Unadjusted and adjusted odds ratios for variables included in the conditional logistic regression model for the scoliosis

Variable	Crude odds ratio (95% CI)	*p* value	Adjusted odds ratio (95% CI)	*p* value
Age	1.28 (1.18–1.38)	< 0.01*	1.17 (1.03–1.34)	0.02*
*Standing posture*				
Normal	ref		ref	
Anterior pelvic tilt	2.36 (1.63–3.40)	< 0.01*	2.73 (1.41–5.28)	< 0.01*
Hunchback	1.88 (1.52–2.33)	< 0.01*	1.27 (0.89–1.80)	0.18
*Sitting posture*				
Straight	0.53 (0.44–0.64)	< 0.01*	0.69 (0.50–0.96)	0.03*
Leaning forward	1.25 (1.07–1.47)	< 0.01*	1.10 (0.84–1.45)	0.48
Leaning left	1.52 (1.27–1.82)	< 0.01*	1.33 (0.97–1.82)	0.08
Leaning right	1.26 (1.02–1.55)	0.03*	0.89 (0.62–1.28)	0.54
Chest touching desk	0.97 (0.79–1.18)	0.76		
Cross-legged	1.05 (0.88–1.25)	0.62		
Others	0.96 (0.65–1.42)	0.84		
*Sleeping posture*				
Supine	ref		ref	
Prone	0.73 (0.53–1.00)	0.05	0.76 (0.44–1.32)	0.33
Left lateral	1.41 (1.15–1.73)	< 0.01*	1.12 (0.81–1.55)	0.48
Right lateral	1.51 (1.22–1.86)	< 0.01*	1.38 (1.00–1.91)	0.05*
*Ever received posture improvement suggestions*				
Yes	ref		ref	
No	0.85 (0.72–1.00)	0.05	1.00 (0.75–1.32)	0.98
*Ever received postural education in school*				
Yes	ref			
No	1.11 (0.93–1.32)	0.26		
*Napping on school desk*				
Yes	ref			
No	1.18 (0.96–1.45)	0.13		
*School desk height*				
=	ref		ref	
<	1.62 (1.34–1.95)	< 0.01*	1.40 (1.04–1.90)	0.03*
>	1.72 (1.32–2.25)	< 0.01*	0.85 (0.54–1.32)	0.47
*Home desk height*				
=	ref		ref	
<	1.60 (1.31–1.95)	< 0.01*	1.29 (0.93–1.78)	0.13
>	1.73 (1.36–2.21)	< 0.01*	1.61 (1.09–2.38)	0.02*
Eyesight*				
Normal	0.89 (0.75–1.04)	0.14		
Myopia	1.04 (0.89–1.23)	0.59		
*Regular change of sitting position in classroom*				
No	ref		ref	
Yes	0.79	0.01*	0.72 (0.53–0.98)	0.04*
*Classroom lighting*				
Appropriate	ref			
Bright	0.84 (0.64–1.10)	0.19		
Dark	1.17 (0.77–1.77)	0.47		
*Schoolbag use habits*				
Backpack on the back	ref			
Backpack on the front	0.68 (0.43–1.10)	0.12		
Shoulder bag	0.84 (0.50–1.41)	0.51		
Cross shoulder bag	0.54 (0.27–1.09)	0.09		
*Shoe wear patterns*				
No difference	ref			
Left shoe wears harder	1.11 (0.79–1.55)	0.56		
Right shoe wears harder	1.13 (0.82–1.56)	0.44		
*Extracurricular classes*				
No	ref			
Yes	0.91 (0.76–1.10)	0.33		
*PE class (per week)*				
None	ref			
1	0.49 (0.12–2.03)	0.32		
2	0.38 (0.10–1.50)	0.17		
3	0.42 (0.11–1.65)	0.21		
> 3	0.49 (0.12–1.96)	0.31		
Average physical activity time (per week, *min*)	0.997 (0.995–0.999)	< 0.01*	1.00 (0.99–1.00)	0.21
Annual height increment, mean (SD), *mm*	0.99 (0.98–1.00)	< 0.01*	0.99 (0.99–1.00)	0.07
*General health status*				
Fair	ref			
Good	0.87 (0.48–1.58)	0.66		
Poor	1.23 (0.70–2.17)	0.47		
*Low back pain*				
No	ref			
Yes	0.91 (0.76–1.08)	0.28		
Sports participation (after school)				
*Badminton*				
No	ref		ref	
Yes	0.83 (0.70–0.98)	0.03*	0.94 (0.71–1.24)	0.66
*Basketball*				
No	ref			
Yes	0.96 (0.78–1.17)	0.68		
*Running*				
No	ref			
Yes	1.02 (0.87–1.18)	0.85		
*Swimming*				
No	ref		ref	
Yes	0.69 (0.57–0.84)	< 0.01*	0.83 (0.58–1.20)	0.33
*Dancing*				
No	ref			
Yes	1.09 (0.80–1.50)	0.58		
*Mountain climbing*				
No	ref		ref	
Yes	0.68 (0.52–0.90)	< 0.01*	0.83 (0.51–1.35)	0.45
*Ping pong*				
No	ref			
Yes	0.79 (0.59–1.05)	0.10		
*Morning exercises*				
No	ref			
Yes	0.79 (0.63–1.01)	0.06		
*Football*				
No	ref			
Yes	0.87 (0.64–1.19)	0.39		
*Stroll*				
No	ref			
Yes	0.50 (0.20–1.24)	0.13		

*Significant differences between groups

We observed statistically significant relationships between postural habits, desk heights and elevated AIS risks. Compared with students who stand normally, those standing with anterior pelvic tilt were 173% more likely to develop AIS (OR = 2.73, 95% CI 1.41–5.28). In terms of sleeping postures, study subjects who slept right laterally had a 38% increase in AIS risk compared with those who slept on their back (OR = 1.38, 1.00–1.91). And after adjusting for other significant factors, inappropriate desk heights, both too low (OR = 1.40, 95% CI 1.04–1.90) and too high (OR = 1.61, 95% CI 1.09–2.38), remained related to increased AIS risks.

Meanwhile, sitting normally and sitting positions in the classroom change regularly were associated with lower odds of AIS. The adjusted ORs are 0.69 (95% CI 0.50–0.96) for sitting normally, and 0.72 (95% CI 0.53–0.98) for sitting positions change.

Sports participations were not significantly associated with AIS in the multivariate regression model.

## Discussion

This study extended the existing literature by establishing relations between desk heights and the risk of AIS for the first time, and producing new evidence of associations between postural habits and AIS risks.

Our study results showed that children who sit at desks that are too high or too low for their heights tend to have elevated risks for AIS. In a previous cross-sectional study exploring lifestyle factors related to AIS [14], table at which participants ate a meal was investigated; however, no significant relations were found between table types and AIS risk. We hypotheses that desks in appropriate sizes help school children maintain good sitting postures and thus are beneficial for them in having reduced AIS risks. An 8-month trial [19] using height-adjustable desks in the classroom confirmed that height-adjustable desks and pedagogical strategies to reduce/breakup sitting bouts could modify schoolchildren’s sitting patterns positively. Therefore, desks and chairs adaptable to individual students’ heights and weights are advised to be introduced and utilized in schools as well as at home. In China, functional sizes and technical requirements of chairs and tables for educational institutions are set by national standard (GB/T 3976-2014). Schoolchildren should have access to desks and chairs that are appropriate for their heights, given that the national standard is strictly followed. However, due to its advisory nature and limited budget, not all schools are equipped with desks and chairs in different sizes as suggested, and schoolchildren tend to use the same set of desk and chair throughout their 3- to 6-year study period, which, for middle school students, is also their growth spurt period. To solve this “one-size for all” class furniture problem, we suggest education authorities, schools and parents consider height-adjustable study desks and chairs for children to sit in the classroom as well as at home. Moreover, according to an analysis of course load of Chinese primary and secondary school students [20], Chinese secondary school students spend an average of 2.2 h on homework assignments every day, indicating prolonged daily sitting time for school children. Although maintaining correct sitting posture has a positive impact on the occurrence of back pain and on reducing AIS risk, it is difficult for schoolchildren to learn and keep a correct sitting posture throughout prolonged sitting periods. Therefore, the introduction of height-adjustable desks should be accompanied by educational programs that include instructions about optimal sitting posture, trainings about adjusting desks to student’s height, as well as classroom posters depicting correct postures, to help children develop and maintain good postures. To further justify introducing adjustable desks and educational programs at school as preventive measures for AIS, economic evaluations assessing the cost-effectiveness of such measures need to be conducted.

Similar to sitting up-straight, students who had their classroom sitting positions change regularly also had a decreased risk for AIS compared with those who at fixed positions. In their 2014 study [21], Drzał-Grabiec and colleagues compared a series of parameters describing body postures and scoliosis among schoolchildren in sitting positions and found that maintaining prolonged sitting position resulted in advanced scoliosis. We hypotheses that changing positions leads to changing distances between students and the front center of the classroom, where students tend to focus on in class, and this change in distance prevents students from adopting certain sitting patterns and decreases the impact of incorrect postures on their musculoskeletal health. This finding indicates that changing students’ sitting positions regularly could be an easy-to-implement and low-cost preventive measure for AIS.

Compared with their counterparts who stand normally, increased odds of AIS were observed in participants who stand with anterior pelvic tilt. This may be related to the lumbar spine in hyperextension and an increased lordotic curvature when standing with anterior pelvic tilt. In their previous study [22], Minghelli et al. found that incorrect standing posture was associated with increased odds of lower back pain. But it is not clear whether incorrect standing posture causes back pain and scoliosis or *vice versa*. Therefore, for now, our finding suggests that during AIS screening, screeners should pay more attention to students with anterior pelvic tilt while standing as they are high-risk individuals, and if applicable, these students should also be considered as the target population for selective screening. In addition, for physical therapists or other clinicians, this finding indicates that exercises targeting standing posture correction, especially anterior pelvic tilt, should be effective in reducing AIS risks.

In the present study, schoolchildren with a right lateral sleeping posture showed an increased risk of AIS compared with those who sleep in supine position. This finding agrees with a 2016 Chinese study [17] that reported an OR of 2.99 for right lateral sleeping individuals, but contradicts with one published by Japanese researchers [14], where lateral sleeping schoolgirls showed a lower AIS risk (OR = 0.86). Since both studies varied in study design, study populations and sample sizes, and these findings were no longer significant after adjusting for other significant variables, comparisons between these results should be with caution.

In terms of sports participation, in their prospective study [23], McMaster and colleagues found that there was an increased odd of AIS in children who were introduced to an indoor heated swimming pool within the first year of life. Similarly, one cross-sectional study [24] carried out by Zaina et al. concluded that participating in competitive sports, such as swimming, was associated with increased risk of trunk asymmetries and hyper kyphosis. In contrast, swimming was not identified as AIS-related factor in either the present study or another large-scale cross-sectional study [14]. Further researches about the swimming frequency, duration of swimming training and the time to start swimming need to be done before conclusions about the impact of swimming on AIS could be made.

Moreover, our study findings implied that human movement may affect the development and onsite of AIS. And movement sciences could contribute to the research and prevention of AIS by investigating the interactions between movement and AIS, and determining the optimal movement patterns, periods and intensity for at-risk population.

Our study has several limitations. Firstly, due to its retrospective nature, the present study is prone to recall bias. Apart from age and sex, other information collected through the questionnaire was self-reported and could not be verified by objective measurements and thus was subject to recall bias and may not fully reflect the facts. In future studies, photographs [25] and other clinical posture assessment tools [26–28] could be utilized to collect objective postural parameters for more accurate posture assessment.

Secondly, we did not perform radiographic examination to confirm the screening findings as it was not feasible for children who were not suspected of AIS to have X-ray scan after screening. Even though according to our screen method validation analysis, the accuracy of the employed screening method was 0.81, our study was limited by the lack of radiographic data for AIS diagnosis confirmation. This could be addressed in future studies by introducing new noninvasive diagnostic tests, such as 3-D ultrasound scan, or developing training programs for screen personnel to improve screening accuracy.

A major strength and innovation of the present study is the inclusion of a series of postural and lifestyle related factors that have never been studied in previous researches, in particular, the desk heights. In addition, the sample size in the present study is by far one of the largest; therefore, the possibility of random variations contributing to present findings is low, and the results are more reliable, compared to small-sample studies.

## Conclusions

In conclusion, inappropriate school desk height, anterior pelvic tilt and lateral right sleeping position are associated with elevated AIS risk, while up-straight sitting and changing classroom sitting positions regularly are associated with decreased AIS risks. These findings could inform future school AIS prevention programs.

## Data Availability

The datasets generated and analyzed during the current study are available from the corresponding author on request.
